# Pazmanniani Physici Ordinarii, Hollandicæ Scientiarum Academiœ, quæ Harlemi est, Membri, Lactucæ Sylvestris contra Hydropem Vires, sive observationum circa morbos acutos et Chronicos factarum

**Published:** 1781-04

**Authors:** 


V. Henrici Jofephi Collin, *Sacr. Cæfareo Reg. Apoft. Majeft. Regimin. infer. Auftr. Confiliarii, et Nofocom. Pazmanniani Phyfici Ordinarii, Hollandicæ Scientiarum Academiæ, quæ Harlemi eft, Membri, Lactucæ Sylveftris contra Hydropem Vires, five obfervationum circa morbos acutos et Chronicos factarum. Pars* vi. 8vo. Viennæ 1780. *pag.* 66. *Cum tabulâ Æn.*

THE plant recommended in this work as a remedy for dropfy, is the *Lactuca Virofa* of Linnæus, or what in this country is vulgarly called *ftrong fcented wild Lettuce.* It is a very common plant in hedges, &c. and flowers in Auguft. It abounds with an acrid, bitter, milky juice, the fmell of which refembles that of opium. We are cautioned not to confound it with the *fonchus oleraceus*, or *common fow thiftle*, which it refembles in the colour of its flowers, which are yellow, and in the fhape of its leaves, which are large and indented with a fpinous rib.

Our

Our author recommends an extract of this plant prepared from its expressed juice, which, after being kept in a cellar, or any other cool place, for four-and-twenty-hours, to prevent its fermenting, is to be strained through a cloth, and clarified with a sufficient quantity of the whites of eggs, after which it is to be again filtered through a flannel bag, and boiled gently till about half of it has evaporated. It is then to be removed from the fire, clarified a second time as before, and after being again filtered, is to be placed over a gentle fire till it has acquired the consistence of an extract. Dr. Collin advises us to gather the plant while in flower, as its virtues are then in the greatest perfection.

Twenty-four cases of dropsy are related, all of which are said to have been cured by the use of this medicine, one only excepted, which proved fatal, but not without the patient's having experienced the use of the extract. In this case, upon dissection, the cavities of the thorax were found full of water, the left lobe of the lungs abounded with hard tubercles, three of which were as large as walnuts; the liver was indurated; the gall-bladder empty; the spleen of three times its usual bulk; the whole of the pancreas schirrous; the colon greatly distended; and the kidneys uncommonly soft.

In

In dropfical cafes, when the difeafe was owing to a general laxity of the folids, our author found that much fmaller dofes of the extract were neceffary than in dropfies of long ftanding, originating from vifceral obftructions. In the former he gave from eighteen grains to half a drachm a day of the extract; in the latter, he prefcribed from one to three drachms in the fame fpace of time. He obferves, that in moft of the cafes he began with fmall dofes. He has conftantly found it a mild remedy, perfectly agreeable to the ftomach. In general it leffened, or altogether extinguifhed the thirft of which the patients had complained before they began to take the extract.

It commonly kept the body open, but without exciting a purging. It feldom failed to promote a copious difcharge of urine, and likewife to act at the fame time as a mild diaphoretic.

All the patients were allowed to drink freely of diluting liquors, while they were taking the extract.

